# Immune Effector Cell‐Associated Hematotoxicity With Secondary Myelofibrosis Following Idecabtagene‐Vicleucel for Multiple Myeloma, Comprehensively Treated With Autologous Hematopoietic Stem Cell Boosting

**DOI:** 10.1002/jha2.70348

**Published:** 2026-07-06

**Authors:** Keishi Ogura, Takahiro Fujino, Takahisa Nakamura, Akihiro Miyashita, Akio Onishi, Taku Tsukamoto, Shinsuke Mizutani, Yuji Shimura, Shinichi Fuchida, Junya Kuroda

**Affiliations:** ^1^ Division of Hematology and Oncology Department of Medicine Kyoto Prefectural University of Medicine Kyoto Japan; ^2^ Department of Blood Transfusion Kyoto Prefectural University of Medicine Kyoto Japan; ^3^ Department of Hematology Japan Community Health Care Organization Kyoto Kuramaguchi Medical Center Kyoto Japan

**Keywords:** autologous hematopoietic stem cell boosting, chimeric antigen receptor T cell (CAR‐T) therapy, immune effector cell‐associated hematopoietic toxicity, multiple myeloma, secondary myelofibrosis

## Abstract

Pancytopenia accompanied by chimeric antigen receptor T cell (CAR‐T) therapy is termed immune effector cell‐associated hematopoietic toxicity (ICAHT), which is often sustained and refractory to supportive care. We report a case of a 52‐year‐old man with multiple myeloma who developed severe ICAHT with secondary myelofibrosis (sMF) after administration of idecabtagene‐vicleucel, which was comprehensively improved by autologous hematopoietic stem cell boosting. This case focuses on sMF as a rare but cautionary complication of CAR‐T therapy, underscoring the importance of bone marrow biopsy to exclude sMF and of stem cell boosting for the treatment of persistent ICAHT.

## 1 Case Report

**FIGURE 1:**
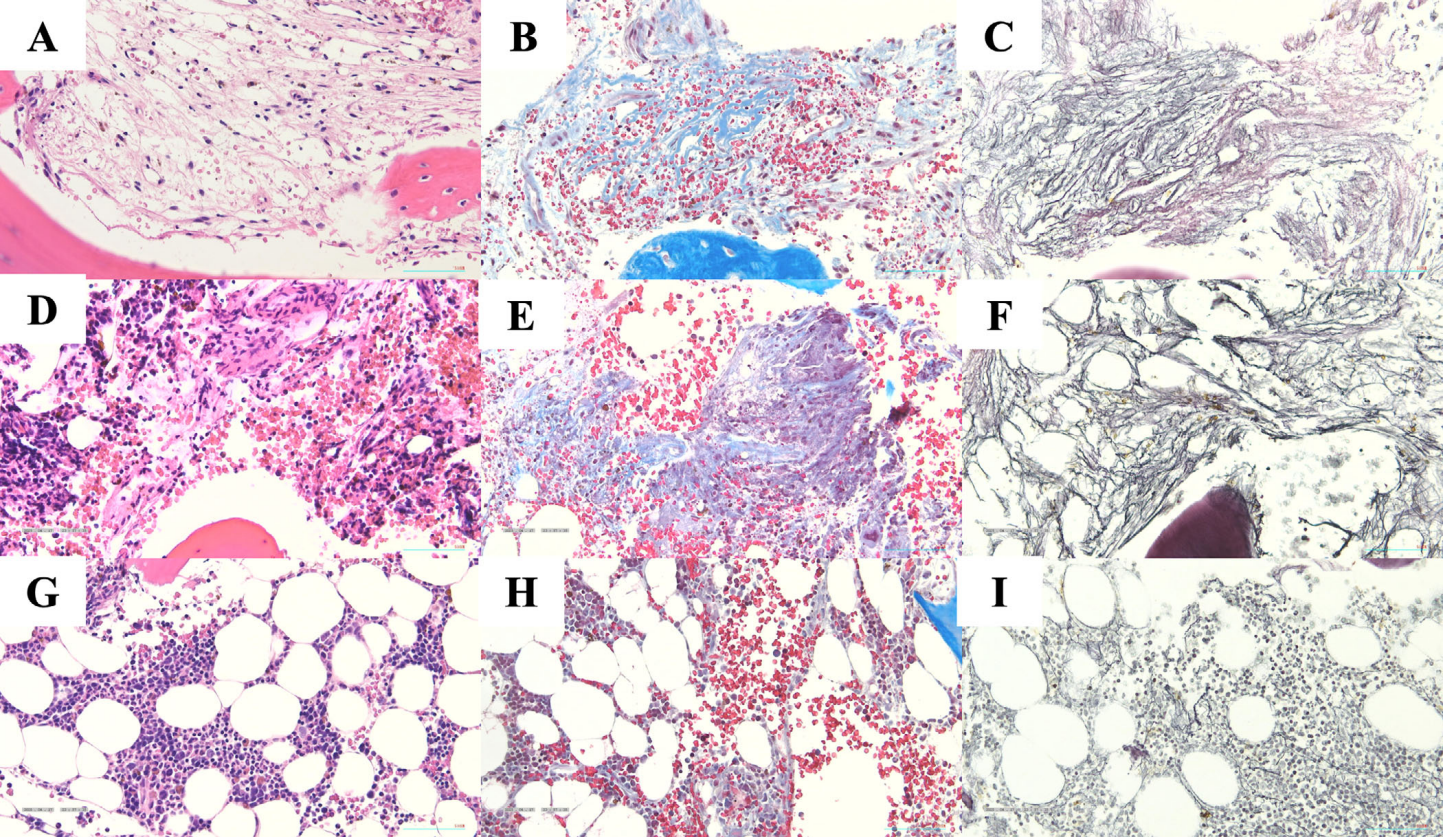
The findings of bone marrow biopsy (×200) using hematoxylin and eosin (H&E), Masson trichrome, and silver staining on Days 25 (A–C), 205 (D–F), and 405 (G–I) post‐CAR‐T infusion, respectively.

**FIGURE 2:**
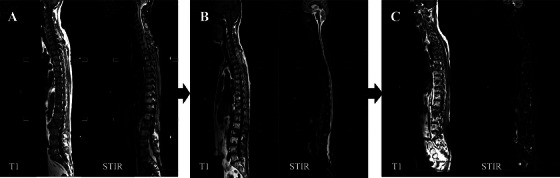
MRI findings of the whole spine, taken 56 days before (A), 26 days after (B), and 140 days after CAR‐T infusion (C), respectively. STIR, short inversion time inversion recovery; T1, T1‐weighted image.

**FIGURE 3:**
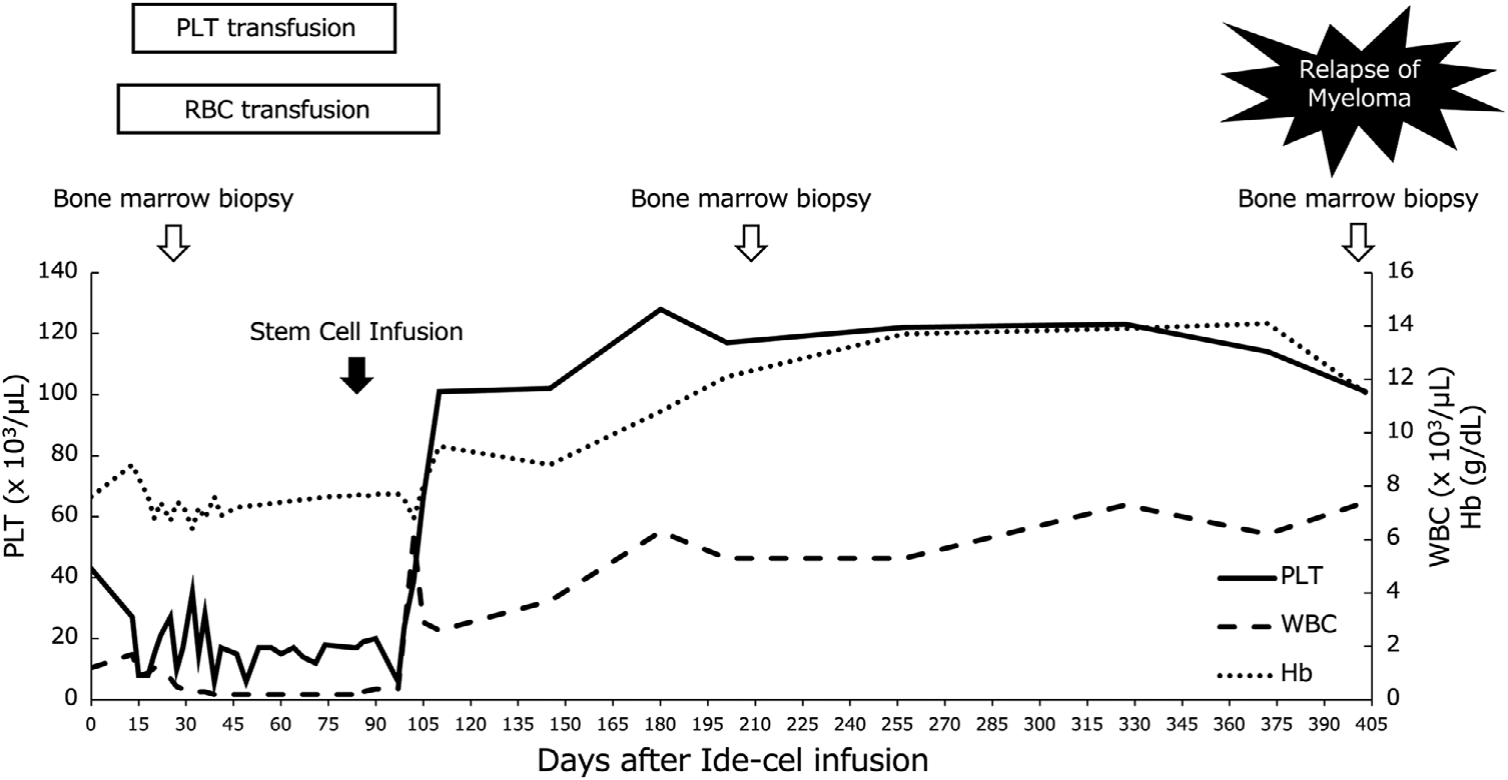
The timeline of blood count, transfusion, and bone marrow biopsy following CAR‐T infusion. Ide‐cel, idecabtagene‐vicleucel; PLT, platelet; RBC, red blood cell; Hb, hemoglobin; WBC, white blood cell.

The patient was a 52‐year‐old male, initially diagnosed with multiple myeloma (MM) of the Bence Jones‐κ type 6 years ago, with chromosomal translocation t(11;14)(q13;q32) and a deletion of 17p. He had been heavily pretreated and repeatedly complicated by multiple bone lesions. Prior treatment included first‐line triplet induction therapy with bortezomib (BTZ), lenalidomide (LEN), and dexamethasone (DEX), followed by high‐dose melphalan and autologous stem cell transplantation with 3.97 × 10^6^/kg CD34‐positive cells. After that, he received consolidative therapy with carfilzomib, LEN, and DEX, followed by LEN maintenance therapy, which kept his disease in complete response (CR) for 2 years according to the International Myeloma Working Group uniform response criteria [1]. After the first relapse, various salvage therapies were administered in sequence, including daratumumab, BTZ, and DEX; elotuzumab, pomalidomide, and DEX; and isatuximab, CFZ, and DEX. Finally, he was indicated for chimeric antigen receptor T cell (CAR‐T) therapy as the fifth‐line treatment and proceeded to lymphocyte collection to produce idecabtagene‐vicleucel (ide‐cel). During the CAR‐T production period, however, the disease progressed aggressively, with the emergence of new extramedullary disease (EMD) originating from the posterior aspect of the L2 lumbar vertebra. Bridging therapy with focal radiotherapy (8 Gy/1 fraction) to the lumbar EMD and systemic chemotherapy with cisplatin, doxorubicin, cyclophosphamide (CPA), and etoposide was performed, resulting in a minor response, and then followed by lymphocyte‐depleting therapy with CPA and fludarabine, then an Ide‐cel infusion. Grade 2 cytokine release syndrome (CRS) occurred on Day 2 but resolved in 4 days with supportive care using tocilizumab and DEX. Simultaneously, Grade 4 pancytopenia occurred and persisted for more than 3 weeks. A bone marrow biopsy performed on Day 25 after ide‐cel infusion revealed increased reticulin fibrosis consistent with Grade 2 myelofibrosis (Figure 1A–C). Megakaryocytes were sparse and therefore lacked the morphological features of primary myelofibrosis, namely, dysmorphic megakaryocytes and abnormal clustering. In addition, we confirmed the absence of splenomegaly and MPN‐related gene mutations in his peripheral blood, including JAK2 V617F, MPL W515L/K, and CALR type 1/2 mutations, suggesting a diagnosis of secondary myelofibrosis (sMF). Three months later, the pancytopenia had not improved, presenting with severe agranulocytosis refractory to G‐CSF and transfusion dependence on red blood cells and platelets. As a solution, we administered autologous peripheral stem cell boosting on Day 85 after ide‐cel infusion. The stem cells had been cryopreserved for 6 years; however, viability was 83% as assessed by trypan blue staining, indicating that CD34‐positive cells amounted to 6.5 × 10^6^/kg. In addition, myeloma cell contamination was ruled out by flow cytometry before injection. As a result, white blood cell counts rapidly increased with short‐term G‐CSF support, followed by a steady increase in red blood cell and platelet counts. Neutrophils, platelets, and red blood cells recovered on Day 13 (> 0.5 × 10^9^/L), Day 18 (> 20.0 × 10^9^/L), and Day 26 (retaining transfusion independence) after stem cell boosting, respectively. Regarding disease control, he achieved a stringent CR on Day 87. Subsequently, he reached a minimal residual disease (MRD)‐negative status on Day 205 after ide‐cel infusion; however, despite recovery of bone marrow function with a nucleated cell count of 55.0 × 10^9^/L with a myeloid/erythroid ratio of 2.7 at this point, Grade 2 myelofibrosis persisted (Figure 1D–F). Regarding magnetic resonance imaging (MRI) of the whole spine, a transition to a fibrotic marrow pattern was confirmed on Day 26 after ide‐cel infusion (Figure 2B), characterized by diffuse high signal on T1‐weighted images and diffuse low signal on short inversion time inversion recovery (STIR), despite a normal hematopoietic pattern having been seen before ide‐cel infusion (Figure 2A). Interestingly, the abnormal MRI findings, as well as bone marrow fibrosis, persisted after hematopoietic recovery on Day 140 post‐ide‐cel infusion (Figure 2C). Notably, the resolution of sMF was finally confirmed on Day 405 after ide‐cel infusion (Figure 1G–I), despite the recurrence of MM being diagnosed at that moment simultaneously (Figure 3).

## 2 Discussion

CAR‐T cell therapies have become integral components of treatment strategies for various hematologic malignancies, contributing to improved patient outcomes, including ide‐cel and ciltacabtagene autoleucel for MM [2, 3]. However, immune effector cell‐associated hematopoietic toxicity (ICAHT) remains an unavoidable and challenging complication in CAR‐T therapy, and unmet needs persist. A predictive model, CAR‐HEMATOTOX, has been developed to estimate the risk of ICAHT, and real‐world data in patients with refractory diffuse large B‐cell lymphoma have demonstrated that those with high CAR‐HEMATOTOX scores are associated with poorer survival outcomes [4, 5, 6].

sMF is considered a rare complication of CAR‐T therapy. In a retrospective study of routine bone marrow biopsies obtained pre‐ and post‐CAR‐T infusion in 259 patients, mainly with malignant lymphoma, 18 patients (7%) were confirmed to have Grade 2–3 myelofibrosis during the peri‐CAR‐T infusion period [7]. Notably, nine of these patients had myelofibrosis at baseline, suggesting that about 3.5% of the cohort developed sMF post‐CAR‐T infusion. Interestingly, patients with myelofibrosis, regardless of whether it was pre‐ or post‐CAR‐T infusion, were reported to have significantly poorer survival in this study, although individual information about recovery from myelofibrosis was not detailed. Furthermore, the underlying biology behind these findings was not clearly elucidated.

To our knowledge, this case report is the first to detail the clinical course of a patient who developed sMF after CAR‐T infusion and achieved resolution 1 year after autologous stem cell boosting. In contrast, there is one prior report of a patient with B‐cell acute lymphoblastic leukemia who developed sMF after Grade 4 CRS following CD19 CAR‐T therapy but did not recover and subsequently died [8]. Although the optimal management of sMF, including both treatment and prevention, remains unknown, our experience suggests that autologous stem cell boosting is an effective modality for managing this rare but severe complication associated with CAR‐T therapy.

The mechanisms underlying sMF following CAR‐T therapy remain unclear. TGF‐β, widely recognized as a key cytokine contributing to primary marrow fibrosis [9], has been reported to be elevated in the inflammatory environment of sMF associated with hematologic neoplasms [10]. However, in the context of CAR‐T therapy, TGF‐β levels were suppressed during CRS after CAR‐T administration, whereas pro‐inflammatory cytokines such as IL‐6, IFN‐γ, IL‐10, and TNF‐α increased significantly [11], casting doubt on the role of elevated TGF‐β in the development of sMF after CAR‐T infusion. In another study assessing prolonged cytopenia after CAR‐T therapy, disruption of the bone marrow niche and increased pro‐inflammatory cytokines were suggested as contributing factors, while the association with sMF remains unelucidated [12]. Therefore, a future step is to conduct cytokine analysis specific to the development of sMF after CAR‐T therapy. Incidentally, MM itself may have the potential to induce sMF [13]; however, this mechanism seems unlikely in the present case, as fibrosis progressed in the MRD‐negative bone marrow after CAR‐T infusion, and improvement was confirmed at the subsequent MM relapse.

Regarding ICAHT management, a multicenter retrospective study of patients with relapsed and refractory MM reported that autologous stem cell boosting achieved hematopoietic recovery in 95% (18/19) of cases [14]. A recent review article also recommended TPO receptor agonists for continuous ICAHT [15]; however, they may be contraindicated in this case because they could accelerate marrow fibrosis as a side effect.

In summary, we successfully managed severe ICAHT with sMF using autologous stem cell boosting in a patient with heavily pretreated MM. A bone marrow biopsy is key to detecting sMF after CAR‐T therapy and should be performed in cases of severe, long‐lasting ICAHT.

## Author Contributions

K.O. and T.F. drafted the manuscript. J.K. reviewed and revised the manuscript. K.O., T.F., T.N., A.M., A.O., and S.F. treated the patient. T.T., S.M., and Y.S. supported the preparation of the draft. J.K. supervised the study. All authors reviewed and approved the final manuscript.

## Funding

The authors have nothing to report.

## Ethics Statement

The authors have nothing to report.

## Consent

The authors obtained signed permission from the patient to publish this case report.

## Conflicts of Interest

Takahiro Fujino has received honoraria from Takeda Pharmaceutical, Bristol Myers Squibb (BMS), Chugai Pharmaceutical, Nippon Shinyaku, AbbVie, SymBio Pharmaceuticals, and Johnson & Johnson Innovative Medicine (J&J). Taku Tsukamoto has received honoraria from AbbVie, Genmab, J&J, BMS, AstraZeneca, Chugai Pharmaceutical, Sanofi, SymBio Pharmaceuticals, Astellas Pharma, and Ono Pharmaceutical. Shinsuke Mizutani has received honoraria from Sanofi, Otsuka Pharmaceutical, Novartis, Nippon Shinyaku, and PharmaEssentia Japan. Yuji Shimura has received honoraria from Ono Pharmaceutical, BMS, J&J, Sanofi, Kyowa Kirin, Takeda Pharmaceutical, and Chugai Pharmaceutical. Shinichi Fuchida has received honoraria from Takeda Pharmaceutical, Sanofi, and J&J. Junya Kuroda is a consultant for J&J, Pfizer, AbbVie, and BMS; has received research funding from Kyowa Kirin, Chugai Pharmaceutical, Asahi Kasei, Sumitomo Pharma, Otsuka Pharmaceutical, Mochida Pharmaceutical, and Japan Blood Products Organization; and has received honoraria from J&J, Kyowa Kirin, Chugai Pharmaceutical, Ono Pharmaceutical, Sanofi, AstraZeneca, Eisai, AbbVie, Novartis, Daiichi Sankyo, Amgen, Otsuka Pharmaceutical, and BMS. The other authors declare no conflicts of interest.

## Data Availability

The data that support the findings of this study are available on request from the corresponding author. The data are not publicly available due to privacy or ethical restrictions.
